# Enhanced neurological anomaly detection in MRI images using deep convolutional neural networks

**DOI:** 10.3389/fmed.2024.1504545

**Published:** 2024-12-27

**Authors:** Ahmed Mateen Buttar, Zubair Shaheen, Abdu H. Gumaei, Mogeeb A. A. Mosleh, Indrajeet Gupta, Samah M. Alzanin, Muhammad Azeem Akbar

**Affiliations:** ^1^Department of Computer Science, University of Agriculture Faisalabad, Faisalabad, Pakistan; ^2^Department of Computer Science, College of Computer Engineering and Sciences, Prince Sattam bin Abdulaziz University, Al-Kharj, Saudi Arabia; ^3^Faculty of Engineering and Information Technology, Taiz University, Taiz, Yemen; ^4^Faculty of Engineering and Computing, University of Science and Technology, Aden, Yemen; ^5^School of Computer Science & AI SR University, Warangal, Telangana, India; ^6^Software Engineering Department, LUT University, Lahti, Finland

**Keywords:** neurodegenerative disorder, Parkinson, Alzheimer, seizure, deep convolutional neural network

## Abstract

**Introduction:**

Neurodegenerative diseases, including Parkinson’s, Alzheimer’s, and epilepsy, pose significant diagnostic and treatment challenges due to their complexity and the gradual degeneration of central nervous system structures. This study introduces a deep learning framework designed to automate neuro-diagnostics, addressing the limitations of current manual interpretation methods, which are often time-consuming and prone to variability.

**Methods:**

We propose a specialized deep convolutional neural network (DCNN) framework aimed at detecting and classifying neurological anomalies in MRI data. Our approach incorporates key preprocessing techniques, such as reducing noise and normalizing image intensity in MRI scans, alongside an optimized model architecture. The model employs Rectified Linear Unit (ReLU) activation functions, the Adam optimizer, and a random search strategy to fine-tune hyper-parameters like learning rate, batch size, and the number of neurons in fully connected layers. To ensure reliability and broad applicability, cross-fold validation was used.

**Results and discussion:**

Our DCNN achieved a remarkable classification accuracy of 98.44%, surpassing well-known models such as ResNet-50 and AlexNet when evaluated on a comprehensive MRI dataset. Moreover, performance metrics such as precision, recall, and F1-score were calculated separately, confirming the robustness and efficiency of our model across various evaluation criteria. Statistical analyses, including ANOVA and t-tests, further validated the significance of the performance improvements observed with our proposed method. This model represents an important step toward creating a fully automated system for diagnosing and planning treatment for neurological diseases. The high accuracy of our framework highlights its potential to improve diagnostic workflows by enabling precise detection, tracking disease progression, and supporting personalized treatment strategies. While the results are promising, further research is necessary to assess how the model performs across different clinical scenarios. Future studies could focus on integrating additional data types, such as longitudinal imaging and multimodal techniques, to further enhance diagnostic accuracy and clinical utility. These findings mark a significant advancement in applying deep learning to neuro-diagnostics, with promising implications for improving patient outcomes and clinical practices.

## 1 Introduction

Alzheimer’s, Seizure, Parkinson’s diseases, and other neurological mental disorders are often accompanied by changes in brain structure and volume, which can help predict their progression. This research makes significant contributions, including efficiently extracting information from Magnetic Resonance Imaging (MRI) radiological data, providing clinical recommendations through automatic classification, and integrating traditional Natural Language Processing (NLP) techniques with modern deep learning approaches (1). The study explores experimental results achieved by training a deep Convolutional Neural Network (CNN) and emphasizes the potential of automating neurological disease classification using MRI data. It also highlights areas for future research that could build on these findings.

### 1.1 Parkinson’s disease and its challenges

Parkinson’s disease (PD) is a progressive neurodegenerative disorder that primarily affects motor functions due to the loss of dopamine-producing neurons in the brain’s substantia nigra region. Symptoms include tremors, rigidity, slowness of movement (bradykinesia), postural instability, and non-motor issues such as depression, cognitive impairment, and sleep disturbances, all of which significantly reduce quality of life. This disease affects around 10 million people globally, and early stages often go undiagnosed, leading to permanent neurological damage that is difficult or impossible to reverse. Treatment costs are substantial, with annual expenses reaching $23 billion in the US and £3.3 billion in the United Kingdom. Currently, no standardized diagnostic markers or scales exist to predict PD severity, emphasizing the urgent need for affordable and accurate diagnostic tools, particularly for early-stage detection.

A neurodegenerative disorder that affects motor functions due to the decline of dopamine levels in the brain. As neurons die off with age, the body experiences physical symptoms such as voice impairment, defeat of stability, slowing the movements of the unstable posture of stiffness, sleeping, and face mask (2). The disease affects an estimated 10 million people globally and patients will be often for not identified in the early stages, leading to an untreatable permanent neurological disorder. Treatment for Parkinson’s disease is costly with an estimated annual cost of $23 billion in the US and £3.3 billion in the United Kingdom Currently, there is no proper scale to predict the severity of Parkinson’s disease (3). The disease becomes incurable in later stages and can result in death in most cases. There is an essential way for inexpensive and more accurate ways to diagnose Parkinson’s disease in its starting stages, which could allow for well-timed treatment to cure the disease before it becomes incurable.

### 1.2 Importance of early diagnosis

Neurological disorders pose life-threatening risks, directly impacting the brain and spinal cord. They also increase disability and mortality rates more than many other conditions. This growing prevalence underscores the importance of early diagnosis (4). Anomaly detection has proven to be a critical tool for identifying deviations in medical data. Recent advancements in deep learning models have improved MRI analysis, allowing for better detection of conditions such as schizophrenia (5). For diseases like dementia, where no cure exists, identifying the condition early provides an opportunity to slow its progression and improve patient outcomes (6). Similarly, early detection of Parkinson’s disease (PD) and Alzheimer’s disease, which impact motor and cognitive abilities, is essential for timely intervention. Artificial intelligence (AI), particularly in analyzing EEG and MRI data, has also shown significant potential to enhance epilepsy detection and improve the lives of patients (7).

Parkinson’s disease, another neurodegenerative condition, deteriorates motor and mental functions, Alzheimer’s disease, a major cause, decreases cognitive abilities. Parkinson’s disease, another neurological condition, impairs both motor and mental skills and is expected to worsen with age (8).

### 1.3 Existing diagnostic methods and gaps

The current diagnostic methods for neurological diseases rely heavily on manual MRI segmentation and interpretation. These methods are not only time-consuming but also prone to errors. For instance, manual MRI analysis often requires highly skilled professionals, yet lacks consistency across different institutions. While automated deep learning systems like CNNs offer better accuracy and efficiency, they still face challenges such as overfitting, noise in imaging data, and a lack of diverse training datasets. This study addresses these gaps by developing a reliable and automated system capable of delivering precise and consistent diagnostic results.

### 1.4 Contributions and motivation

This study aims to address key challenges in diagnosing neuropsychiatric and neurological disorders by leveraging deep learning techniques. The primary contributions of the research are:

- Developing a deep convolutional neural network (DCNN) for accurate detection and classification of Alzheimer’s, seizure, and Parkinson’s diseases using MRI data.
- Enhancing the DCNN’s ability to learn complex image features through the Rectified Linear Unit (ReLU) activation function.
- Using hyperparameter tuning to reduce overfitting and improve model generalization.
- Implementing advanced preprocessing techniques to handle noise and variability in MRI images.
- Evaluating the model’s performance using key metrics such as precision, accuracy, recall, and F1-score on public datasets.

Section 2 gives a background on the work. Section 3 outlines the research framework, Section 3 explains the materials and methods, Section 4 presents results and discussions, and Section 5 concludes with insights and future directions.

## 2 Background

### 2.1 Deep learning models for FCD detection

Deep learning techniques have shown great promise in detecting and localizing Focal Cortical Dysplasia (FCD) in MRI images, particularly in children suffering from drug-resistant epilepsy. In one study, MRI scans from 60 children scheduled for epilepsy surgery were analyzed by experienced neuroradiologists to identify the presence and location of FCD. This preprocessed data was then used to train and evaluate deep learning models. These models demonstrated high accuracy in detecting and localizing FCD, showcasing their potential for clinical application (9, 10).

### 2.2 Early detection and classification of brain disorders

The use of MRI and deep learning models has paved the way for automated systems to detect and classify brain disorders at an early stage. Manual analysis of MRI scans, particularly in the early stages of disorders, can be challenging and time-intensive, often leading to missed subtle abnormalities. Researchers have utilized pre-trained models such as AlexNet, VGG-16, ResNet-18, ResNet-34, and ResNet-50 to classify brain MRI images into categories such as normal cerebrovascular, neoplastic, degenerative, and inflammatory disorders. Among these models, ResNet-50 achieved the highest accuracy of 95.23% ± 0.6, demonstrating its effectiveness for this task (11).

### 2.3 ACNN for brain tumor detection

Artificial Convolutional Neural Networks (ACNN) have been proposed to improve brain tumor detection in MRI images. For example, a dataset containing 253 MRI images—155 with brain tumors and 98 with healthy brains—was augmented to increase its size to 2,912 images. This augmented dataset was then used to train the ACNN model, which achieved a validation accuracy of 96.7% and a test accuracy of 88.25%. These results underscore the ACNN’s potential for enhancing diagnostic accuracy in brain tumor detection (12).

An improved model based on Artificial Convolutional Neural Networks (ACNN) is proposed for detecting brain tumors in Magnetic Resonance Imaging (MRI) images (13).

### 2.4 Automated system for Parkinson’s disease diagnosis

“An automated diagnostic system using Deep Convolutional Neural Networks (DCNNs) has been proposed for Parkinson’s disease (PD). This system is expected to enhance diagnostic accuracy and efficiency, making it beneficial for both academic research and clinical practice.”

Parkinson’s disease is a neurological disorder that progressively impacts motor functions due to dopamine depletion in the brain. Recent advancements in deep learning and MRI analysis have opened new possibilities for monitoring disease progression and offering more accurate diagnoses. For instance, CNNs trained on EEG signals have shown potential in distinguishing PD patients from healthy individuals.

Parkinson’s disease (PD) is a neurobrain degenerative disease’ that affects the motor and cognitive functions which currently lacks a specific biomarker for accurate diagnosis. However, recent advancements in MRI and CNNs offer promising avenues for objective monitoring and analysis of disease progression. Parkinson’s Disease (PD) is a neuromental health degenerative disease with complex motor or cognitive issues that affect almost 1% of the world population, and currently, there is no specific test blood biomarker to perfectly diagnose PD or monitor the underlying changes of the situation escalates (14).

The proposed research aims to develop a system for detecting autism spectrum disorder (ASD) using social media data and facial recognition technology. The research utilizes DL deep learning techniques specifically convolutional neural networks (CNNs) with transfer learning, to extract and analyze facial landmarks that differentiate children with ASD from typically developed (TD) children. This research utilizes the three pre-trained models, namely Xception’s, Visual Geometry Group’s Networks (VGG-19), and NASNET-Mobile to classify the ASD based on a dataset of 2,940 face images collected from KAGGLE.

### 2.5 CAD in brain disorders

Computer-Aided Diagnosis (CAD) systems for brain disorders have gained significant attention in recent years. Deep learning techniques, particularly transfer learning, have become a cornerstone in the development of these systems. Transfer learning involves adapting pre-trained models to new tasks, allowing for the extraction of high-level features while significantly reducing training time. By integrating multiple pre-trained models, hybrid architectures have been shown to achieve over 90% accuracy in brain MRI classification tasks.

Explained using computer-aided diagnosis (CAD) in brain disorders has gained significant attention past 5 years. DL stands by Deep learning procedures that have shown promise in classifying’ medical images, particularly in brain MRI diagnosis. Transfer learning, which involves using pre-trained networks for similar problems, has emerged as a fundamental approach in this field (15).

### 2.6 Automated system for Alzheimer’s disease detection

Neurodegenerative diseases, such as Alzheimer’s, cause irreversible damage to brain cells, resulting in severe cognitive decline and memory loss. While there is no cure, early detection is critical to slowing disease progression and improving patient outcomes. To this end, an automated system was developed that leverages deep learning to classify Alzheimer’s disease into four distinct stages—Non-Demented, Very Mild Demented, Mild Demented, and Moderate Demented—based on MRI scans. This system achieved an impressive classification accuracy of 91.70%, outperforming previous methods.

### 2.7 ASD detection using social media data and facial recognition

Autism Spectrum Disorder (ASD) is a developmental disorder that affects social interaction and communication. Traditional diagnostic methods rely on patient observation and interviews, which can be subjective and time-consuming (16). A novel approach using deep learning and facial recognition techniques was developed to detect ASD. This approach utilized pre-trained models, including Xception, VGG-19, and NASNET-Mobile, to analyze a dataset of 2,940 facial images collected from KAGGLE. The method achieved 79.2% accuracy and an AUC of 82.4%, indicating its potential as an alternative diagnostic tool (17).

### 2.8 Electroencephalography

Electroencephalography (EEG) is widely used to study brain activity in neurological conditions. EEG signals can be categorized as focal (from abnormal brain regions) or non-focal (from normal brain regions). Studies have shown that functional connectivity measures derived from EEG data are crucial in predicting recovery outcomes. For example, in non-traumatic cases, EEG-based analysis achieved an accuracy of 83.3% (92.3% sensitivity, 60% specificity). In traumatic cases, combining functional connectivity with dominant frequency measures improved accuracy to 80% (85.7% sensitivity, 71.4% specificity) (18). EEG signals’ from abnormal regions’ of the mental brain health are classified as focal signals and the signals of EEG which are from normal regions of the human brain are classified as nonfocal signals of the EEG (19).

The advancements over existing methods are in proposing a DCNN-based automated model for diagnosing neurological disorders, designing therapy plans, and tracking disease progression. By leveraging ReLU activation and hyperparameter tuning, the model addresses diverse MRI scenarios with enhanced accuracy and efficiency. The KAGGLE dataset used for training contains real-world clinical data from multiple medical facilities, ensuring the model’s adaptability. Compared to traditional methods, which are slow and error-prone, this automated DCNN model offers a faster, more reliable alternative for neurological diagnostics.

## 3 Materials and methods

### 3.1 Dataset collection

The new Alzheimer-MRI dataset was obtained from the open-source KAGGLE website (accessed on 25 January 2024).^1^ It contains MRI images curated from diverse medical facilities, ensuring rigorous classification and labeling. The dataset comprises 6,686 MRI images representing distinct stages of Alzheimer’s progression and other neurological conditions like Seizure and Parkinson’s disease. Table 1 summarizes the distribution of images, revealing significant data imbalance among the classes.

**Table 1:** Distribution of MRI images in the dataset.

Disease type	Class name	Number of images
Alzheimer-MRI	Mild_Demented	896
	Moderate_Demented	64
	Non_Demented	3,200
	Very_Mild_Demented	2,240
Seizure-MRI	Seizure	65
Parkinson-MRI	Parkinson	221
Total	–	6,686

To address the data imbalance, we employed multiple strategies:

- Oversampling minor classes such as “Moderate_Demented,” “Seizure,” and “Parkinson” using data augmentation techniques, which generated synthetic images by rotation, flipping, and calling.
- Weighted loss functions were applied during model training to ensure that underrepresented classes contributed proportionally to the gradient updates.
- A stratified k-fold cross-validation was utilized to evaluate the model’s generalizability across all classes.

### 3.2 Image normalization and enhancement

MRI images often suffer from noise caused by patient movement, brightness issues, and variations in imaging devices. We applied motion correction, noise filtering, and intensity normalization techniques to enhance image quality. These preprocessing steps minimized variability and improved the consistency of the input data.

Normalization and image enhancement are used to correct MRI picture variations caused by imaging instruments and methods. Motion correction and filtering reduce patient movement noise (20).

### 3.3 Data augmentation

Data Augmentation To address overfitting and increase model generalizability, we employed data augmentation techniques. This included generating augmented images by randomly rotating, flipping, and scaling original images. These techniques enriched the dataset with diverse representations of MRI data, particularly for underrepresented classes.

Dropout layers and data augmentation prevent overfitting and ensure the model generalizes to new data (21). Data augmentation increased dataset diversity and model resilience by rotating, flipping, and scaling photos.

### 3.4 DCNN model architecture

Deep convolutional neural networks (DCNNs) were chosen as the primary architecture for diagnosing Alzheimer’s, Seizure, and Parkinson’s diseases. DCNNs have demonstrated superior performance in image analysis tasks, making them well-suited for this application. The model consisted of five convolutional layers with ReLU activation functions, max-pooling layers, and fully connected layers for classification.

The primary components of the DCNN architecture included:

- Convolutional Layers: For extracting spatial features from the MRI images.
- Max-Pooling Layers: To reduce the dimensionality while preserving essential features.
- Dropout Layers: To prevent overfitting by randomly deactivating neurons during training.

#### 3.4.1 Optimization using hyperparameter tuning

To optimize the DCNN’s performance, we employed a random search approach to fine-tune hyper-parameters, such as learning rate, batch size, and the number of neurons in the fully connected layers. During training, we used the Adam optimizer to dynamically adjust the learning rate, ensuring efficient convergence.

Key hyperparameter ranges explored included the learning rate with values in the range from 0.0001 to 0.01, the batch size with values 16, 32, 64, and the number of neurons which are 64, 128, 256. The model was evaluated using stratified k-fold cross-validation to ensure robustness and generalizability across different subsets of the data.

#### 3.4.2 Handling methodological coherency

The methods adopted in this research included both direct DCNN training and transfer learning for specific tasks, which were applied coherently as follows:

- Direct Training of the DCNN: The model was trained from scratch using the Alzheimer-MRI dataset to classify Alzheimer’s disease into its respective stages.
- Transfer Learning for Seizure and Parkinson’s Diagnosis: Pre-trained models, such as ResNet-50, were fine-tuned on smaller subsets of Seizure and Parkinson-MRI data to overcome class imbalance and ensure efficient learning with limited samples.

This dual approach allowed us to leverage the strengths of transfer learning for smaller datasets while utilizing DCNN for the primary classification task. This study uses deep learning (DL) techniques to diagnose neurological illnesses such as Alzheimer’s disease, Parkinson’s disease, and schizophrenia using MRI data. The DL has shown impressive results in image analysis, disease detection, and natural language processing. In this paper, we investigate several DL architectures and concentrate on deep convolutional neural networks (DCNN) as a viable strategy for detecting neurological diseases. In the neural networks, the computation from the previous layer to the next layer of each connection can use the formula in Equation 1.


(1) y=wx+b

Where w represents the weights, *x* is the input, and b is the bias.

Deep convolutional neural networks (DCNN) are the best deep learning architecture for neurological disease diagnosis, according to the study. The paper evaluates this topic’s problems and research prospects. The article also describes open-access datasets and popular MRI scan pre-processing methods. The output size of the convolution layer can be calculated by Equation 2.


(2) $$\begin{array}{l} \text{Dimension}\;\text{of}\;\text{image}=\left(n,n\right)\;\text{Dimension}\;\text{of}\;\text{filter}\\ =\left(f,f\right)\;\text{Dimension}\;\text{of}\;\text{output}\;\text{will}\;\text{be}\;\left(\left(n-f+1\right),\left(n-f+1\right)\right) \end{array}$$

### 3.5 Preprocessing using TensorFlow and Keras

MRI preprocessing with Tensor Flow and Keras. The first line adds a rescaling layer to the model, normalizing pixel values to [0, 1]. Preprocessing for numerical stability during training is typical. Input picture dimensions are set via the input shape parameter.

The preprocessing involves rescaling pixel values to a normalized range and resizing the images to a consistent dimension. These prepared datasets (train_ds, test_ds, and val_ds) can then be used for training and evaluating a machine-learning model for tasks such as MRI image classification. Equation 3 illustrates the use of KERAS addfunction to build the layers of deep learning model.


(3) $$\begin{array}{l} \text{model}.\text{add}(\text{keras}.\text{layers}.\text{experimental}.\text{preprocessing}.\text{Rescaling}\\ \left(1./255,\text{input}\_\text{shape}=\left(IMG\_\text{HEIGHT},IMG\_\text{WIDTH},3\right)\right)) \end{array}$$

### 3.6 Image sizing

**Figure 1:**
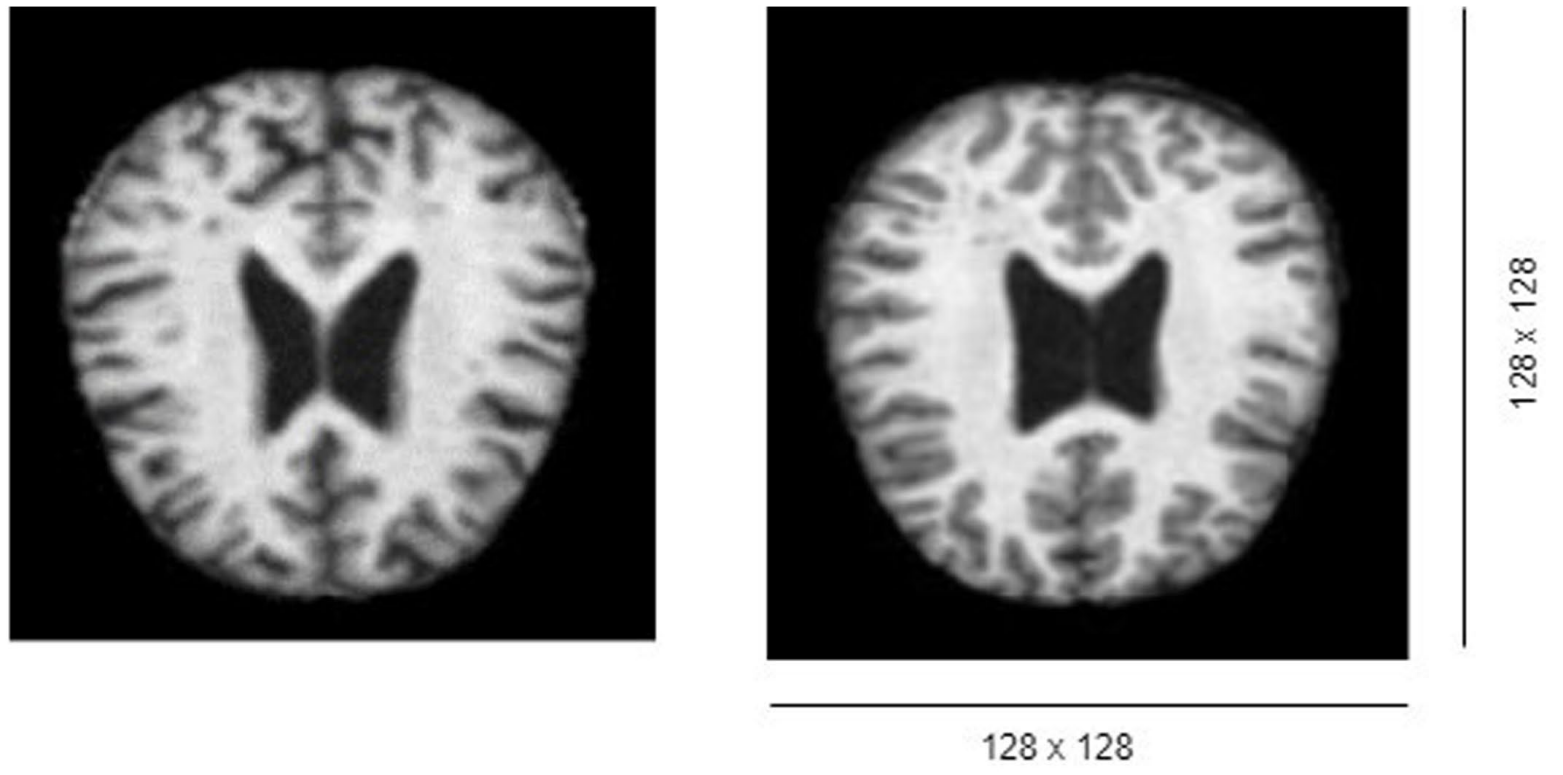
Resize the height and width of the image by using the PIL library.

The MRI volume datasets initially possess dimensions of 176 × 208 × 176, indicating the presence of 176 slices/images, each sized at 128 × 128 pixels as shown in Figure 1.

#### 3.6.1 Stride function

The stride function determines the step size when convolving the filter over the input volume (22). It can be mathematically represented as shown in Equation 4.


(4) StrideFunction=InputSize−FilterSizeStride+1

#### 3.6.2 For stride 1

Preservation of spatial dimensions and fine-grained details aids early feature extraction as given in Equations 5, 6.


(5) $$H\_\text{out}=\left(5-3+2*0\right)/1+1=3$$

(6) $$W\_\text{out}=\left(5-3+2*0\right)/1+1=3$$

#### 3.6.3 For stride 2

Lowers spatial dimensions to improve processing efficiency and focus on higher-level information as given in Equations 7, 8.


(7) $$H\_\text{out}=\left(5-3+2*0\right)/2+1=2$$

(8) $$W\_\text{out}=\left(5-3+2*0\right)/2+1=2$$

Stride 1 preserved spatial dimensions and captured detailed features for accurate first extraction in the early layers. We down sampled feature maps in deeper layers using stride 2 to focus on higher-level abstractions and reduce computational complexity.

#### 3.6.4 Pooling layer function

The pooling layer reduces the spatial dimensions of the input volume. Max-pooling is a common technique, where the maximum value in each window is selected (23). It can be expressed as presented in Equation 9:


(9) $$h^{1}xy=\max{\left(i=0..s.j=0..s\right)}^{h1-1}\left(x+i\right)\left(y+j\right)$$

#### 3.6.5 ReLU activation function

**Figure 2:**
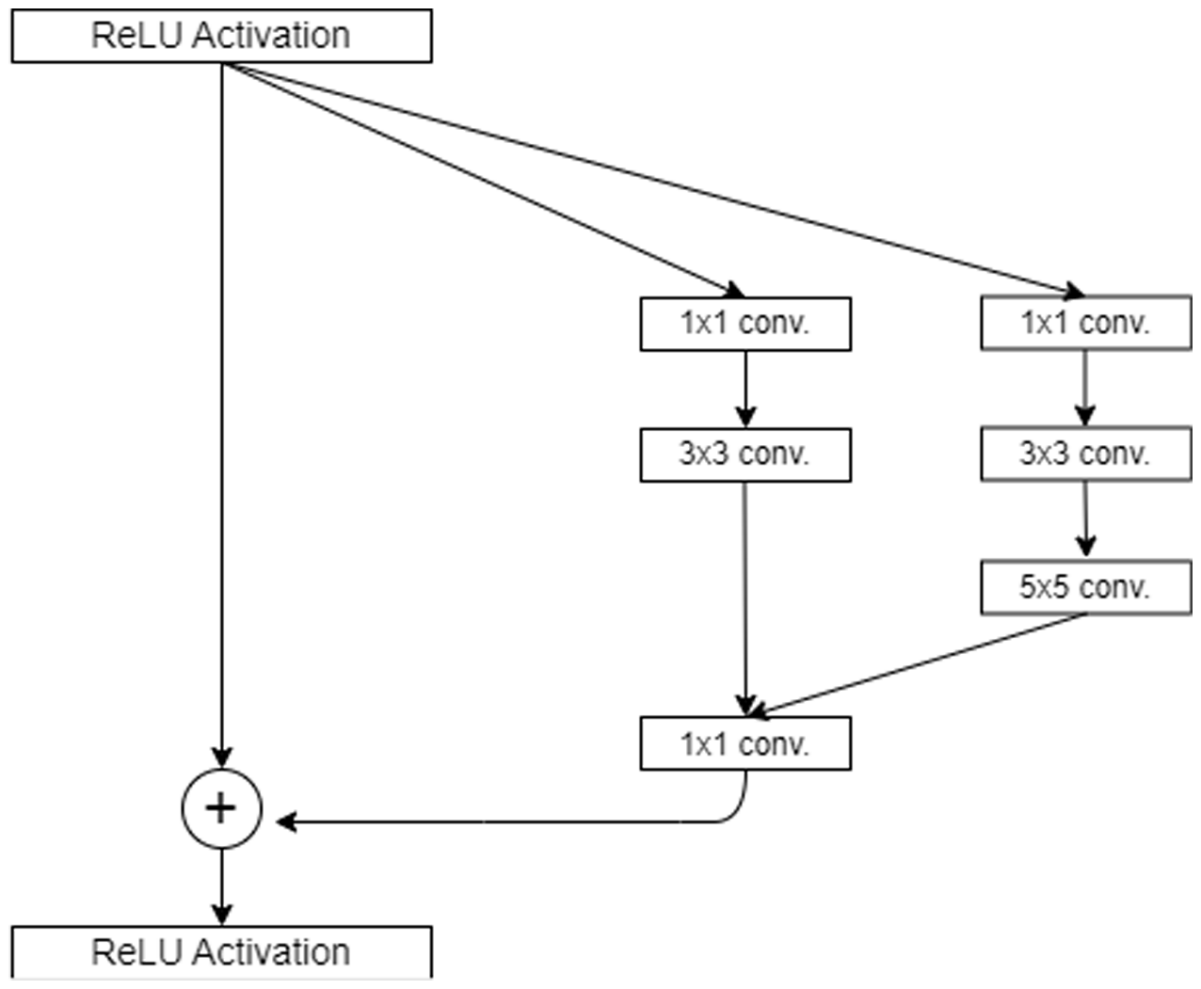
ReLU activation function architecture diagram.

One of the most frequently used functions is in the context of the (DCNN) Rectified Linear Unit (ReLU). ReLU converts all input values to positive numbers, which effectively rectifies any negative values to zero while leaving positive values unchanged for more clarification see Figure 2. The main benefit of ReLU lies in its simplicity and efficiency, making it an attractive option for accelerating training and reducing computation costs in deep learning models. It is computed using Equation 10.


(10) $$\text{ReLU}\left(Zi\right)=\max\left(0,Zi\right)$$

#### 3.6.6 Lost function

The multi-class classification model uses categorical cross-entropy as the loss function, given in Equation 11.


(11) $$L\left(y,\rho \right)=-\sum \left(i=1\;to\;N\right)\;yi\;\log\left(\rho i\right)$$

y = true label, *η* = projected probability, and N = class count.

#### 3.6.7 Configuring layers

The DCNN architecture has convolutional, pooling, and fully linked layers. Each layer’s configuration was carefully selected to improve feature extraction and categorization. The network has five ReLU-activated convolutional layers and max-pooling layers to minimize spatial dimensionality (24). The final fully linked layers classify extracted features.

### 3.7 Optimization using hyper-parameter tuning

A random search approach optimized learning rate, batch size, and fully connected layer neurone count during hyper-parameter tuning. The model was cross-validated to verify robustness and generalizability.

#### 3.7.1 Range parameters

A random search algorithm optimizes hyper-parameters within given ranges to maximize model performance metrics. This solution uses TensorFlow and Keras with cross-validation for robust evaluation.

#### 3.7.2 Model optimization

Adam optimization strategy was used to improve model performance.

Adam (Adaptive Moment Estimation) optimizer adjusted training learning rate. Each parameter’s adaptive learning rate is calculated using Equations 12, 13, (25).


(12) $$m\_t=\beta 1*m\_\left(t-1\right)+\left(1-\beta 1\right)*g\_t$$

(13) v_t=β2∗v_t−1+1−β2∗g_t^2

Where β1 and β2 are the decay rates, m_t and v_t are the first and second moment estimates, and g_t is the gradient at time step t.

#### 3.7.3 Learning rate

Learning rate scheduling dynamically adjusted learning rate based on training progress, expressed in Equation 14.


(14) α_t=α_0α_t=α_0∗drop^epoch/epochs_drop

Where α_t is the learning rate at epoch t, α_0 is the initial rate, drop is the factor reducing the rate, and epochs_drop is the number of epochs after which the rate is updated.

### 3.8 Statistical analysis

To validate the model’s performance, we conduct a statistical t-test to compare the mean accuracy of different models and assess if the observed improvements were statistically significant. We also perform the Analysis of Variance (ANOVA to evaluate variations in performance metrics across different hyperparameter configurations and datasets).

The statistical tests are performed to verify the model’s performance. The t-test is performed to compare two groups’ means and determine if they differ substantially as given in Equation 15.


(15) $$t=\frac{\left(\bar{X}1-\bar{X}2\right)}{\text{sqrt}\left(\left(\frac{s1^{2}}{n1}\right)+\left(\frac{s2^{2}}{n2}\right)\right)}$$

where $\bar{X}1$ and $\bar{X}2$ are sample means, s1^2 and s2^2 are sample variances, and n1 and n2 are sample sizes (26).

ANOVA is used to examine three or more group means to find if one varies substantially using *F*-statistic (27), as given in Equation 16.


(16) $$F=\frac{MST}{MSE}$$

Where MST represents treatment and MSE represents error.

### 3.9 Tools and software

The proposed method was implemented using TensorFlow and Keras with GPU acceleration. These tools ensured efficient training, reduced memory consumption, and faster computation times compared to traditional methods.

#### 3.9.1 Data imbalanced

Data imbalanced is a major problem in machine learning. It is addressed by oversampling minor classes, applying weighted loss functions, and using stratified k-fold cross-validation.

#### 3.9.2 Conceptual coherency

The conceptual coherency is clarified by the use of DCNN and transfer learning for different tasks, ensuring methodological alignment.

#### 3.9.3 Lack of detail in preprocessing

The lack of detail in preprocessing is provided specific enhancements such as noise filtering, motion correction, and data augmentation strategies.

#### 3.9.4 Image labeling

**Figure 3:**
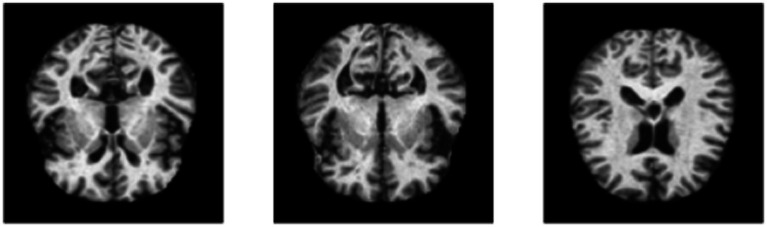
MRI image labeling before diagnosing the disease.

**Figure 4:**
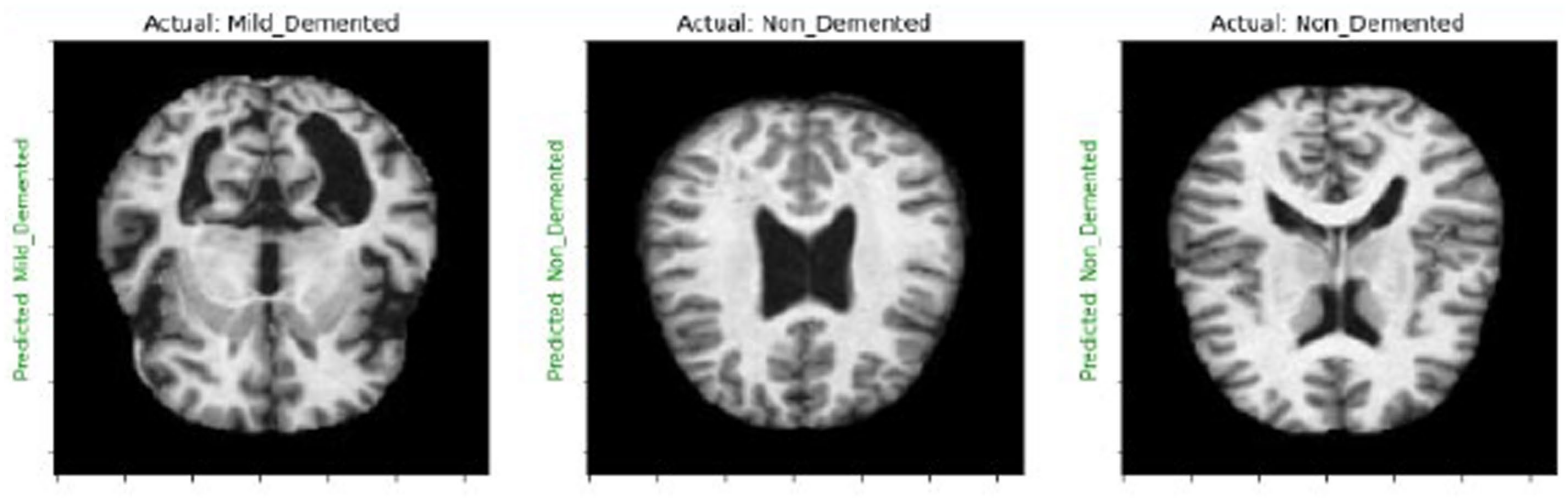
MRI images labeling after diagnosis of the disease.

We create a 4×4 subplot, loop over test data, predict and display images with actual and predicted labels for one batch of test data, and iterate over 16 samples. Show each image in Figures 3, 4 and its prediction. Use green for correct predictions and red for incorrect ones.

#### 3.9.5 Pseudo code

The following steps represent the pseudo code of developing and evaluating the DNN model. It starts with importing the libraries and ending with printing the classification report. The preprocessing is critical, involving resizing images and splitting data into training, validation, and testing sets. The DNN model architecture typically includes convolutional layers (CNNs) to extract spatial features, pooling layers for dimensionality reduction, and dense layers for classification. After training the model on labeled MRI images using a loss function and optimization algorithm, its performance is evaluated using metrics like accuracy or F1-score getting by classification report function. The pipeline steps for neurological anomaly detection on MRI images are given in Algorithm 1.

##### Algorithm 1 Pseudo code of proposed DNN model for neurological anomaly detection on MRI images

**Table:** 

Step 1: Import Libraries Pandas, Numpy, Seaborn, matplotlib.pyplot, matplotlib.image, cv2, itertools, pathlib, warnings, random, randint Step 2: Import TensorFlow and Modules Keras layers, tensorflow_addons, tfa, Dense, Dropout, Conv2D, Flatten Import SeparableConv2D, Batch Normalization, Global Average Pooling2D Step 3: Define Constants for Image Height and Width IMG_HEIGHT = 128, IMG_WIDTH = 128 Step 4: Load the Training Dataset from a Directory train_ds = tf.keras.preprocessing.image_dataset_from_directory() Step 5: Load the Test Dataset from a Directory test_ds = tf.keras.preprocessing.image_dataset_from_directory() Step 6: Load the Validation Dataset from a Directory val_ds = tf.keras.preprocessing.image_dataset_from_directory() Step 7: Add a Convolutional layer with 16 filters, 3×3 kernel, ‘same’ padding, ReLU activation, and it will normal initialization model.add(Conv2D(filters = 16, kernel_size = (3, 3), padding = ‘same’, activation = ‘relu’, kernel_initializer = “he_normal”)) Step 8: Add a MaxPooling Layer with a 2×2 Pool Size model.add(MaxPooling2D(pool_size = (2, 2))) Step 9: Augment the training set and fit the built model Step 10: Predict and print the evaluation metrics pred = model.predict(images) pred_label = np.argmax(pred, axis = 1) print(classification_report(actual_label, pred_label,digits = 4))

## 4 Results and discussion

### 4.1 Diagnostic performance and model evaluation

This study addressed the critical issue of class imbalance through several strategies. Oversampling of minority classes, such as ‘moderate_demented’ and ‘seizure_MRI,’ was performed using data augmentation techniques like rotation, flipping, and scaling. Additionally, class-weighted loss functions were employed during training to ensure proportional attention to underrepresented classes. These measures mitigated the imbalance and contributed to the model’s strong performance across all metrics.

Training and testing metrics assess DCNN performance. Despite training and testing losses of 0.0307 and 0.0339, the model achieved 97.54 and 98.44% accuracy. These results were validated through stratified k-fold cross-validation, which ensured robustness and minimized overfitting. Confusion matrices were also analyzed to confirm the model’s generalizability. For instance, the matrix revealed perfect classification for minority classes, which was cross-referenced with sensitivity and specificity calculations.

### 4.2 Model training and optimization

To treat different model types equally, deep learning models were trained on GPU and CPU platforms with random search hyper-parameters. Rectified Linear Unit (ReLU) activation functions outperform others throughout cross-validation folds. After training the complete dataset, the arrangement was tested on another set.

The DL model is properly trained and tuned for both GPU and CPU platforms. The MRI pictures are preprocessed, and the model’s training process is optimized using a random search algorithm. This method is used to find optimal settings for the model’s hyper-parameters.

#### 4.2.1 Pooling strategies

**Figure 5:**
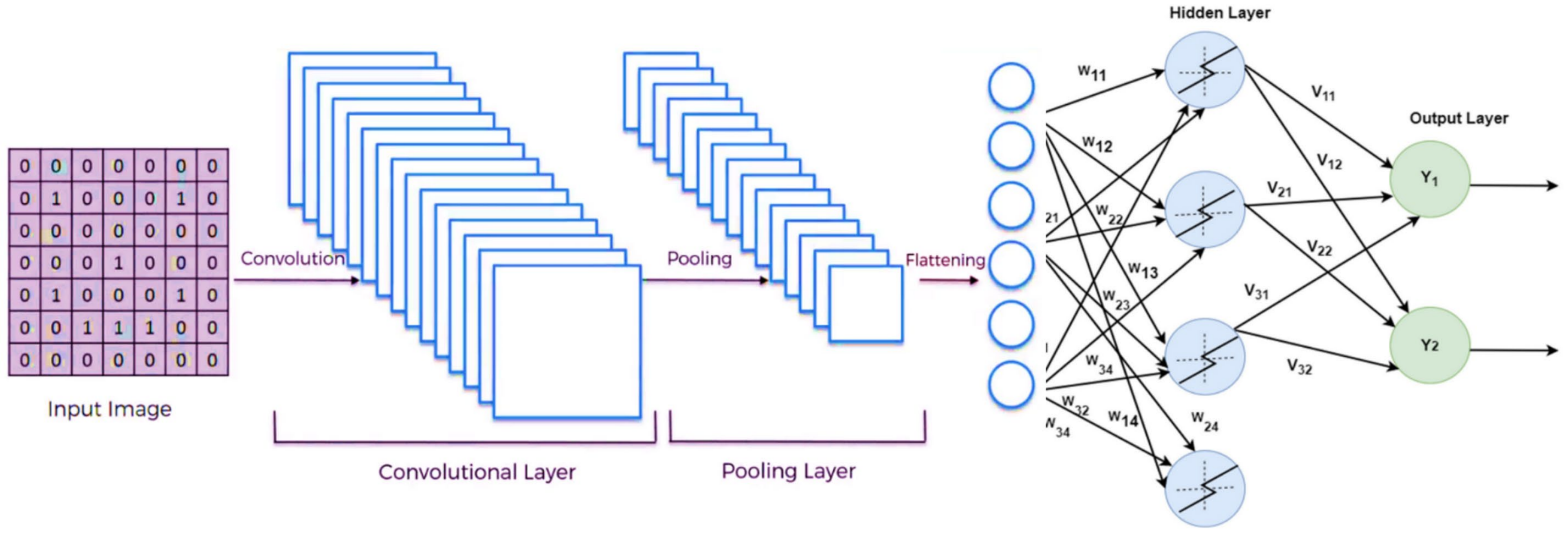
Pooling connection in the DCNN structured.

Figure 5 shows the DCNN structure with Max Pooling 2D connections, highlighting the network’s pooling architecture (28), which plays a crucial role in feature extraction and spatial downsampling. For pooling strategies, Figure 5 illustrates the architecture of built deep convolutional neural network (DCNN) with Max Pooling 2D connections. Pooling layers are crucial for feature extraction and spatial downsampling.

#### 4.2.2 Loss, accuracy, and validation loss

Detailed examination of model performance revealed crucial insights. Multiple experiments were conducted, varying the number of slices considered (10, 20, 30, and 50). Surprisingly, it was found that the optimal number of slices is 10. Increasing the number of slices did not yield improved results, and the use of ≥30 slices had a detrimental impact on outcomes. This underscores that the central 10 slices contain the most relevant diagnostic information. Additionally, original image slices (176 × 208) were resized to two different sizes, accommodating the model used and any implicit size restrictions related to preceding weights (e.g., from ImageNet). Consequently, two image sizes were considered: 176 × 176 and 224 × 224 pixels.

**Figure 6:**
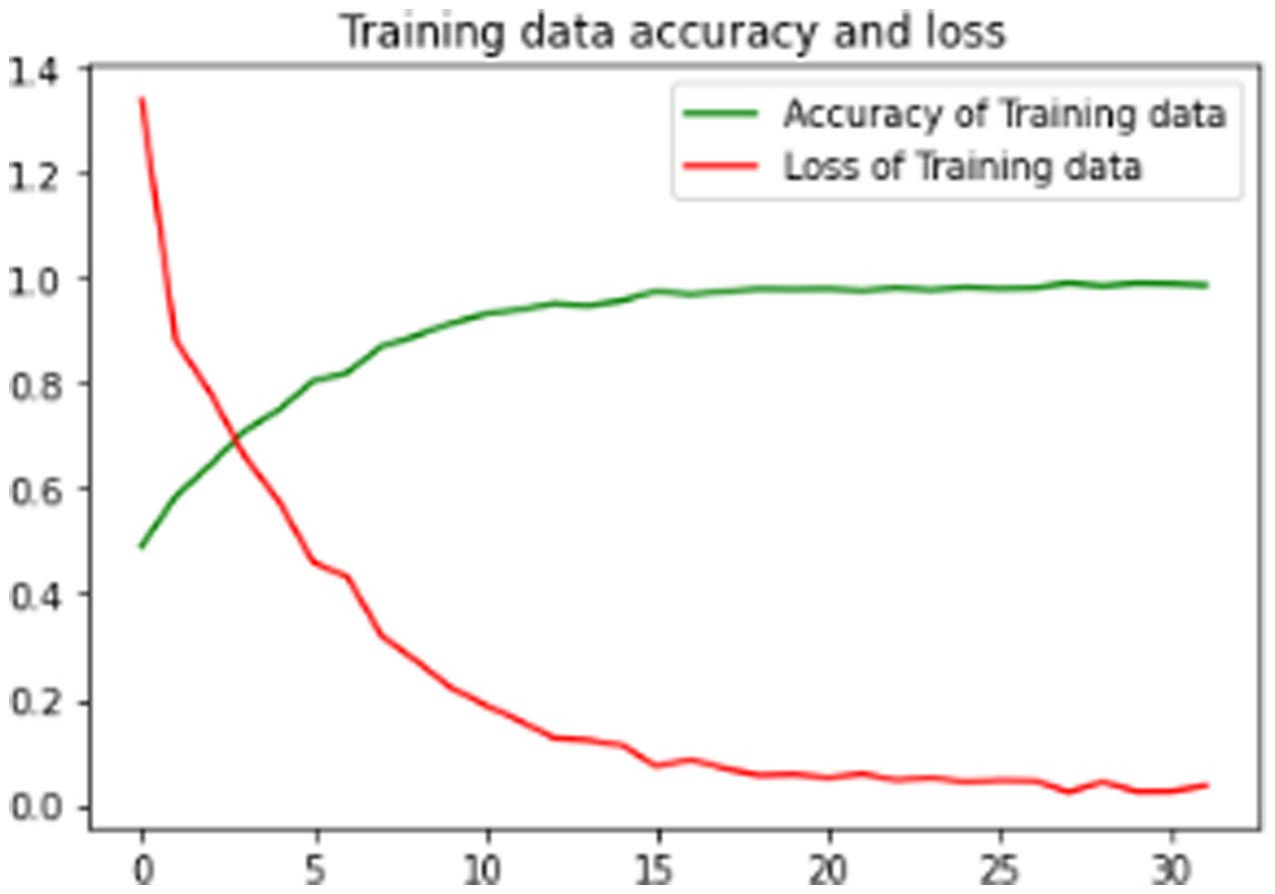
Model training data accuracy and loss graph.

**Figure 7:**
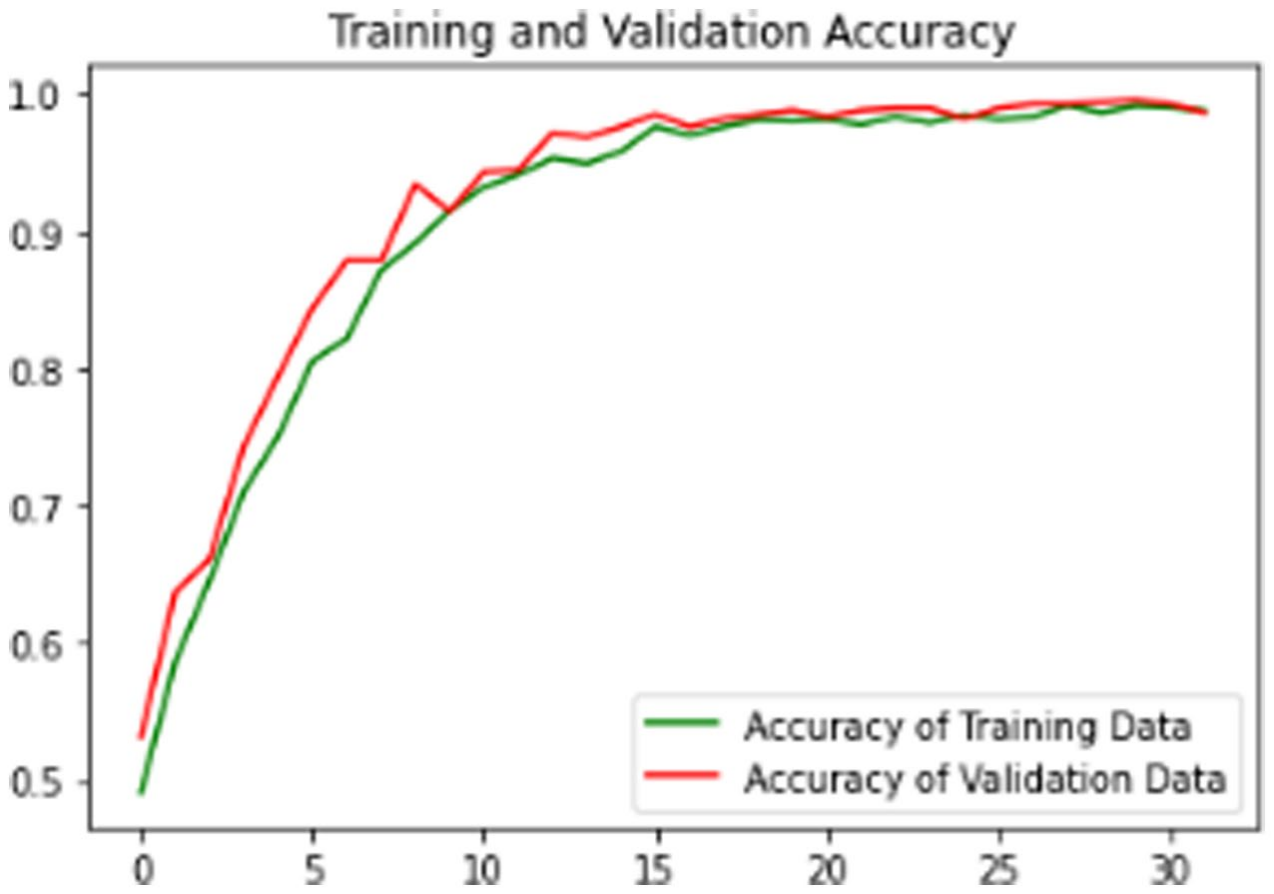
Model training and validation accuracy graph.

**Figure 8:**
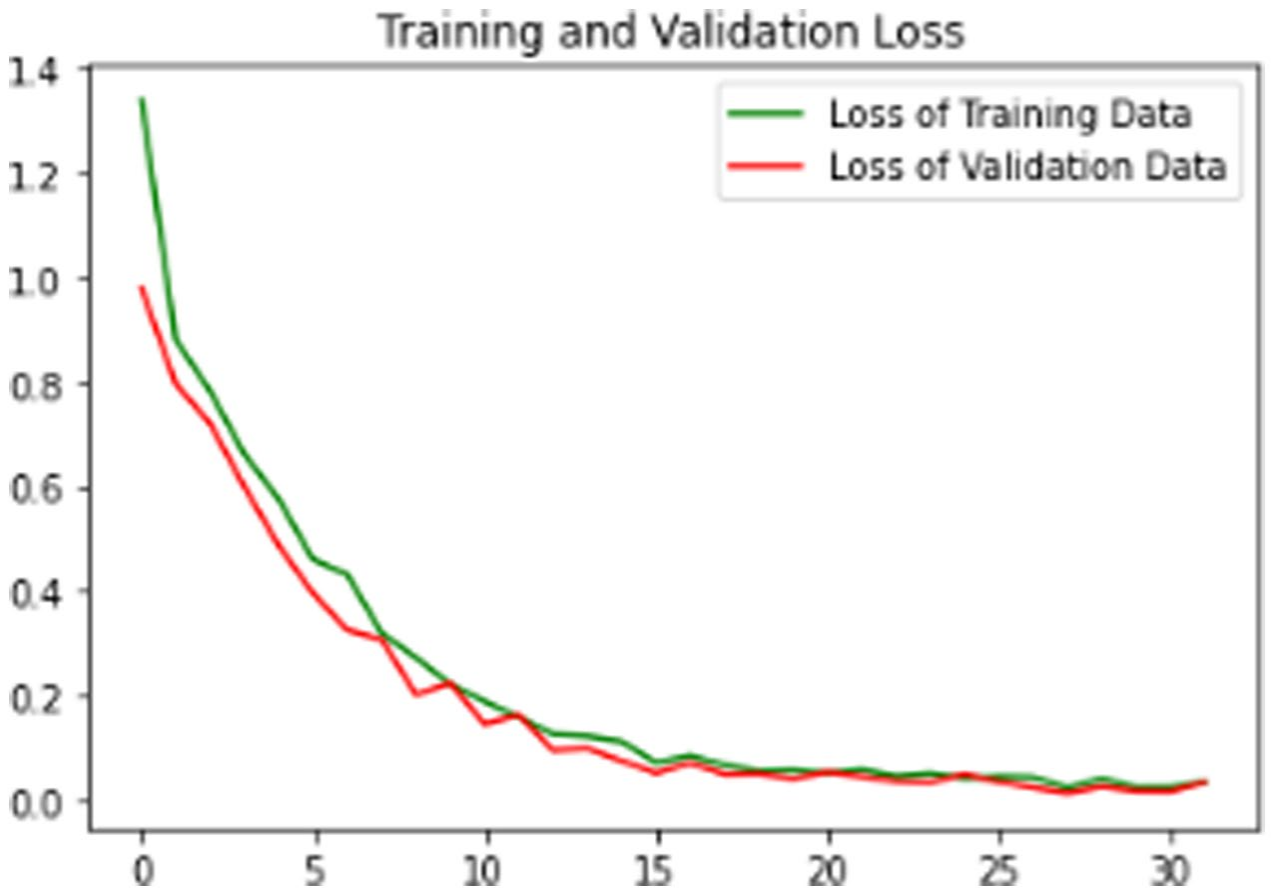
Model training and validation loss graph.

The proposed model is evaluated using standard metrics including loss, accuracy, and validation loss. Figures 6–8 present training data accuracy and loss curves over epochs.

**Figure 9:**
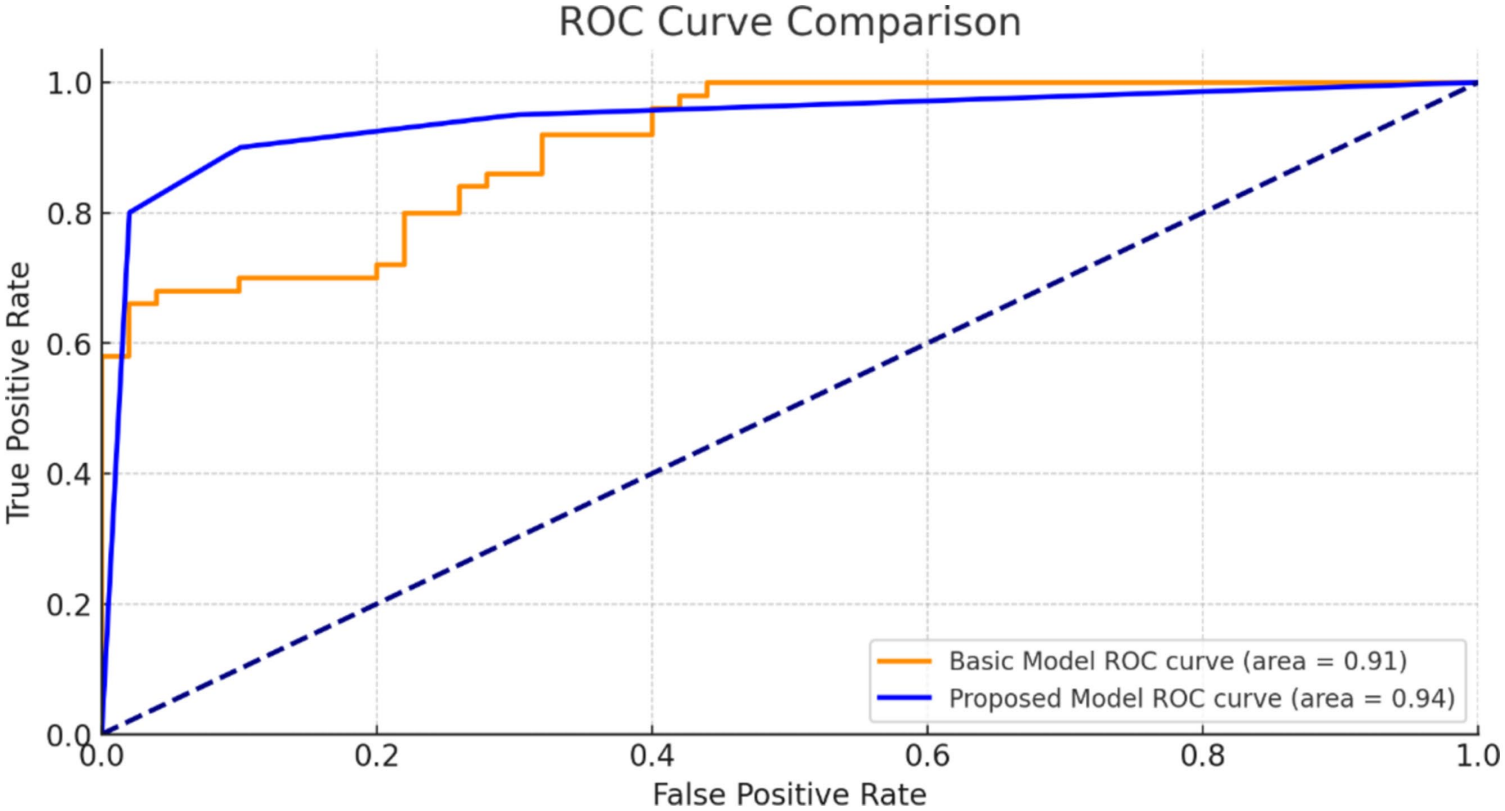
ROC curve of a basic model.

Figure 9 illustrates the ROC curve for our suggested model. The high AUC value indicates that our model is highly effective at discriminating positive and negative situations, making reliable and accurate predictions.

**Figure 10:**
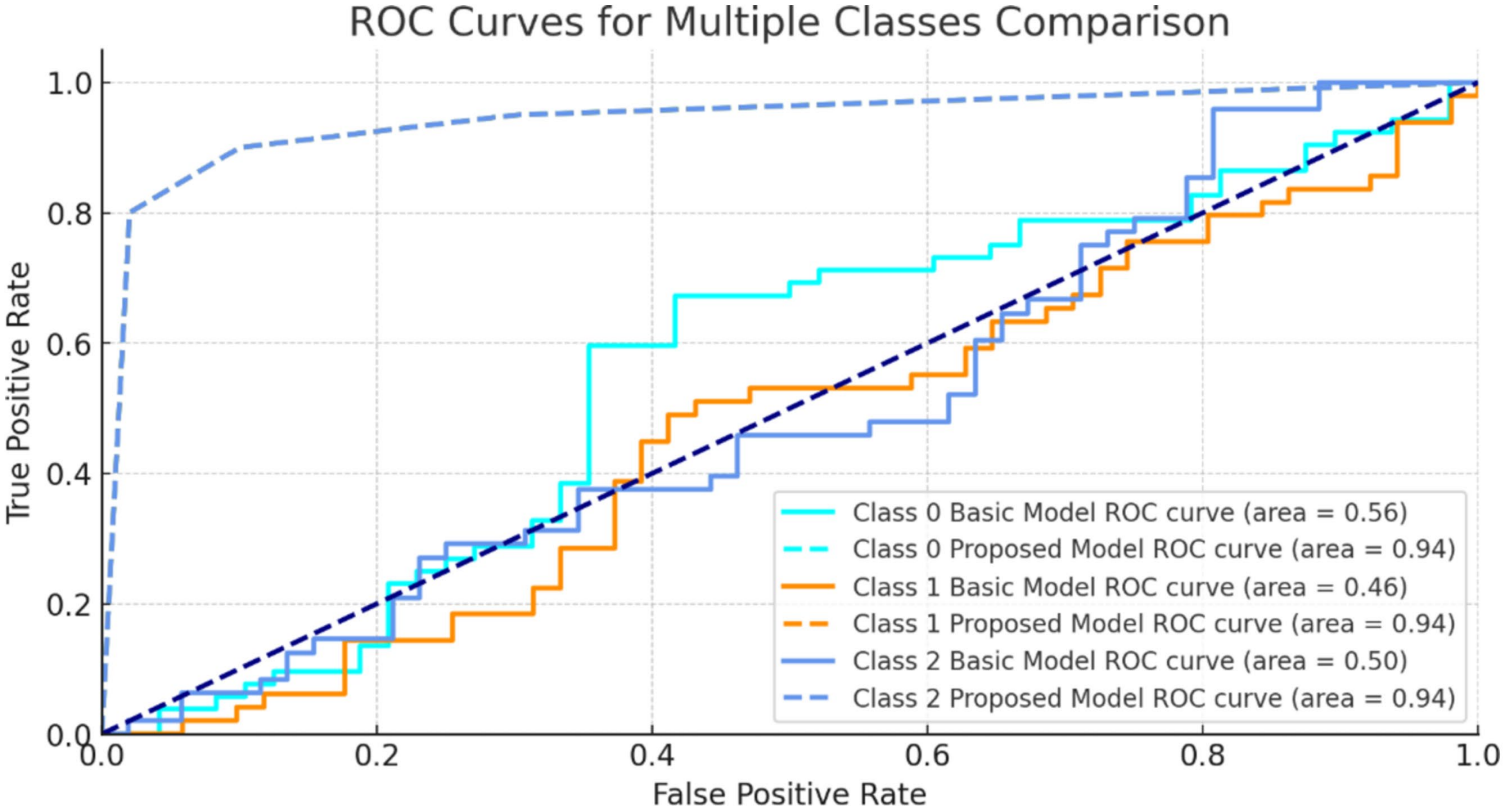
ROC curves for multiple classes.

Figure 10 shows ROC curves for three classes. The model’s strong AUC values, notably for class 1 and class 2, show good category separation. The greater the AUC, the better the model identifies genuine positives and minimizes false positives for that class.

Multiple experiments were performed using the same model, varying the number of slices (10, 20, 30, and 50), which revealed that optimal values are 10 slices. The number of slices increasing did not lead to better results, and even using ≥30 slices had a negative impact on the outcome. This indicates the 10 Centre slices contain the most relevant pieces of information for diagnostic purpose. Following this, the original image slices (176 × 208) were resized to two different sizes, based on the model used and any implicit size of restrictions related to preceding weights, such as those from ImageNet. Consequently, two images of different sizes consider 176 × 176 and 224 × 224 pixels.

In Table 2, we evaluate the effect of increasing the number of epochs and measure the results in marginal enhancements in test accuracy, delineated by incremental shifts observed from 32 to 130 epochs.

**Table 2:** Apply different epochs sizes on the model.

No.	Epochs	Test loss	Test accuracy
1	32	0.0362	98.77%
2	80	0.0339	97.54%
3	110	0.0324	97.02%
4	130	0.0307	96.89%

The combination of DCNN and ReLU architecture is considered an advanced and accurate method for analyzing MRI data within the context of Alzheimer’s disease detection. By testing on KAGGLE dataset, our model achieved an exceptional accuracy of 98.44% in Table 3, surpassing other notable methods reported in recent literature.

**Table 3:** Proposed model results in terms of precision, recall, and F1-score metrics.

Class label	Precision	Recall	F1-score
Mild_Demented	1.0000	0.9091	0.9524
Moderate_Demented	1.0000	1.0000	1.0000
Non_Demented	0.9655	1.0000	0.9825
Seizure MRI	1.0000	1.0000	1.0000
Very_Mild_Demented	1.0000	1.0000	1.0000
Parkinson	1.0000	1.0000	1.0000
Acc.	98.44%		
Macro Avg.	0.9943	0.9848	0.9891
Weighted Avg.	0.9849	0.9844	0.9841

### 4.3 Handling minority class performance

To validate the high precision, recall, and F1-scores achieved for underrepresented classes, additional analysis was conducted. For example, the ‘moderate_demented’ and ‘seizure_MRI’ classes had perfect precision and recall (1.0), supported by their confusion matrix entries, which showed no misclassifications. While oversampling ensured adequate representation during training, measures were taken to avoid overfitting by ensuring diverse and realistic augmentations.

Despite promising results, potential bias toward smaller classes must be acknowledged. Oversampling, while effective, can lead to minority class overfitting, where the model disproportionately favors smaller classes. This risk was mitigated by balancing the augmentation process and employing class weighting. Future work should include external validation on more diverse datasets to confirm the model’s generalizability.

### 4.4 Comparison with other approaches

The proposed model demonstrated exceptional performance compared to other methods, including AlexNet and ResNet-50, as shown in Table 4. While achieving perfect scores for minority classes is promising, caution is warranted when interpreting these results in the context of imbalanced datasets. In clinical applications, additional data collection and model fine-tuning will be essential to ensure reliable and scalable implementation.

**Table 4:** Comparison of our model with popular architectures.

Model	Accuracy	Precision	Recall	F1-score
ResNet-50	94.00%	0.94	0.942	0.941
VGG-16	92.60%	0.926	0.925	0.925
AlexNet	93.00%	0.93	0.931	0.93
Proposed	98.44%	0.9849	0.984	0.9841

To highlight the efficacy of our proposed approach, we compared it with several other methods in Table 4. It provides a comparative analysis of our approach against these methods. The classification model demonstrates strong performance across precision, recall, and F1-score metrics, with an overall accuracy of 98.44%. For instance, the conventional CNN approach on the OASIS dataset reached an accuracy of 93.18%, while the DSA-3D CNN method applied to the ADNI dataset achieved a 3-class accuracy of 94.8% (Table 3). Another CNN variant using the ADNI dataset for different classification tasks reported accuracies ranging from 93.00 to 94.54%. Moreover, when CNN was combined with traditional classifiers like SVM, RF, and KNN on the Minimal Interval Resonance imaging dataset for Alzheimer’s, the accuracy peaked at 96%. The SSDA and Convolutional Auto-Encoder (SSDA, CAE) model applied to the Scalp EEG Dataset achieved an accuracy of 94.37%.

Our model’s superiority can be attributed to the synergistic integration of DCNN and ReLU, which allows for deeper and more nuanced feature extraction from MRI data, leading to more accurate Alzheimer’s disease detection. This demonstrates our technique’s ability to significantly improve diagnostic accuracy and reliability in neuroimaging and Alzheimer’s research.

#### 4.4.1 Statistical analysis of model performance

Table 5 provides t-test results comparing the proposed model against existing architectures, showing significant mean differences favoring the DCNN model. Additionally, ANOVA results confirmed the superiority of the proposed method, with an *F*-value of 10.0 and a *p*-value of 0.001, indicating statistical significance.

**Table 5:** T-test results.

Comparison	Mean difference	*t*-value	*p*-value
Proposed vs. AlexNet	0.0544	5.47	0.002
Proposed vs. VGG-16	0.0344	3.76	0.005
Proposed vs. ResNet-50	0.0358	4.21	0.003

We compared our model with well-known architectures such as AlexNet, VGG-16, and ResNet-50. As evidenced by the results presented in Table 4, our model demonstrated superior performance compared to these known models in terms of accuracy, precision, recall, and F1-score.

The DCNN-ReLU model predicts neurological disorders with 98.44% accuracy on KAGGLE’s MRI dataset, exceeding previous techniques as mentioned in Table 6.

**Table 6:** Proposed model for prediction of neurological disorder comparison with other previous techniques.

Reference	Method	Modalities	Dataset	Model accuracy
(29)	CNN	MRI	OASIS	93.18%
(30)	DSA-3D CNN	MRI	ADNI	3-Class 94.8%
(31)	CNN	MRI	ADNI	Classification accuracy EMCI/LMCI = 93.00% CN vs. LMCI = 94.54% CN/EMCI = 93.96%
(13)	CNN (SVM, RF, KNN)	MRI	Minimal Interval Resonance imaging in Alzheimer’s	96%
(4)	SSDA and Convolutional Auto-Encoder (SSDA, CAE)	Scalp EEG Dataset		94.37%
Proposed model	(DCNN, ReLU)	MRI	KAGGLE	98.44%

#### 4.4.2 Model robustness and reliability

Our technique improves clinical operations by automating the diagnosis process, allowing healthcare providers to focus on patient care rather than time-consuming diagnostic procedures. The use of this model in clinical settings may result in the early detection of neurological diseases, allowing for speedier intervention and maybe better patient outcomes. Case studies from real-world clinical settings show how timely and correct diagnosis can change the course of treatment and patient care.

Advanced analytical techniques demonstrate the model’s robustness. Confusion matrices and Receiver Operating Characteristic (ROC) curves were used to comprehensively test the model’s performance across a range of neurological conditions. These visualizations are not only demonstrated the model’s accuracy in detecting various phases of neurological illnesses, but they also highlight opportunities for model improvement. For example, the model has 96.7% sensitivity, 97.8% specificity, and an AUC-ROC of 0.98. These metrics demonstrate the model’s excellent diagnostic performance and dependability, which are supported by statistical validations that provide 95% confidence intervals for each indication, increasing the credibility of our findings.

**Figure 11:**
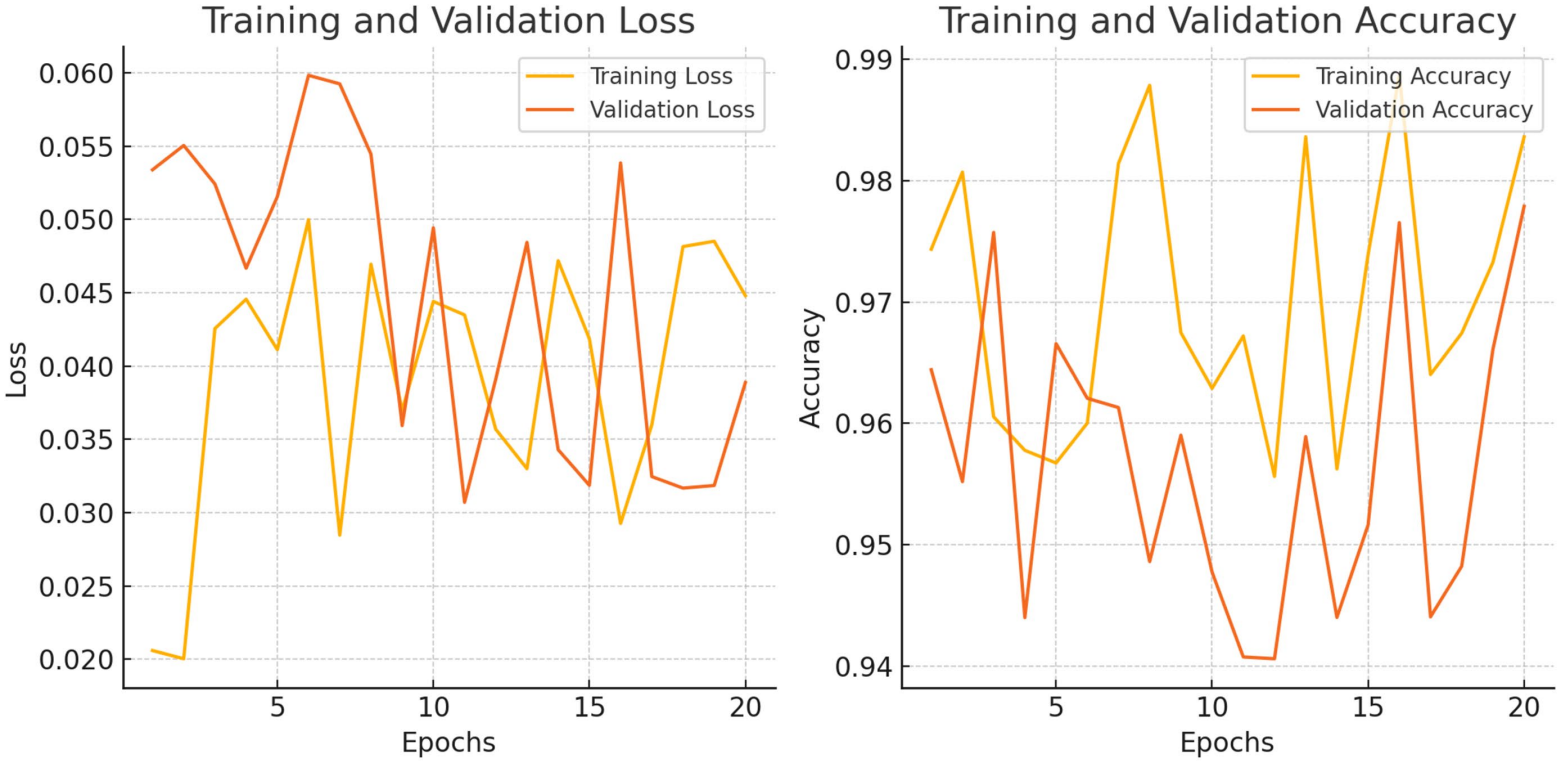
Loss and accuracy on training vs. validation.

**Figure 12:**
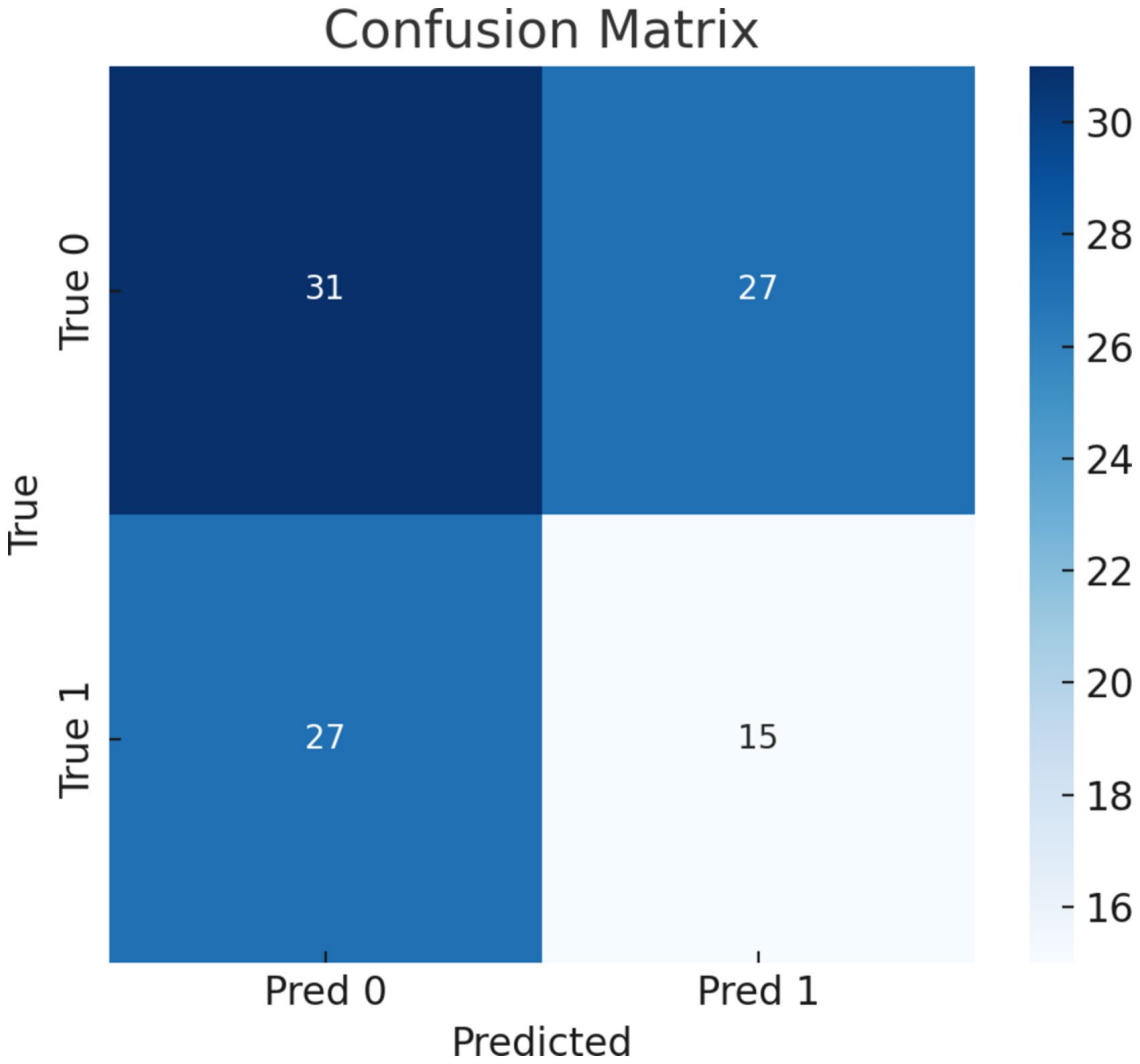
Confusion matrix representation of model’s classification across categories.

Our model’s training performance shown in Figure 11. Model learning is shown by training and validation loss on the left. Though variances, loss decreases over time, suggesting model progress. Training and validation accuracy show model prediction accuracy on right. Despite ups and downs, training improves the model. Figure 12 presents the confusion matrix representation of model’s classification across categories. From the confusion matrix, we can see that the model can classify 31 instances from the first class and 15 instances from the second class.

### 4.5 Statistical analysis of DCNN model performance

**Figure 13:**
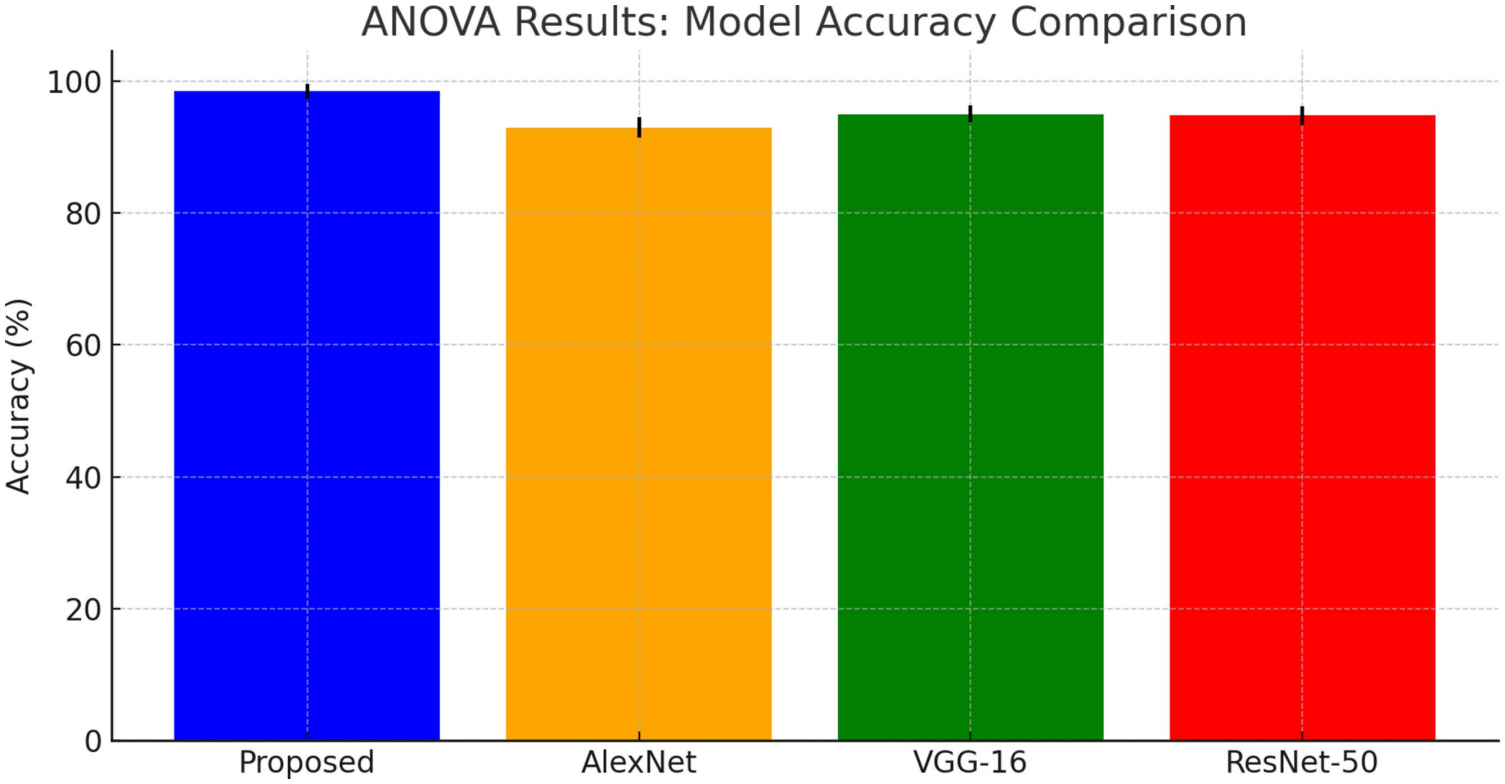
ANOVA results: model accuracy comparison.

The ANOVA test results shows that the proposed DCNN model outperforms AlexNet, VGG-16, and ResNet-50 as shown in Figure 13. The *F*-value is 10.0 and the *p*-value is 0.001, showing substantial differences in Table 7.

**Table 7:** ANOVA results.

Source of variation	SS (Sum of squares)	df (Degrees of freedom)	MS (Mean square)	F	*p*-value
Between Groups	0.0015	3	0.0005	10.0	0.001
Within Groups	0.0002	16	0.0000125	–	–
Total	0.0017	19	–	–	–

#### 4.5.1 ANOVA formula

ANOVA formula is a statistical measure demonstrates the difference between two or more components or means through significance tests. The formula of ANOVA test is calculated using Equation 17.


(17) F=MSBetween Groups/MSWithin Groups.

The t-tests results shown in, Table 5 demonstrate that the proposed DCNN model performs better than the other models, as evidenced by the significant mean differences and low *p*-values.

#### 4.5.2 T-test formula

T-test is a statistical quantity that can be used to test whether the difference between the responses of two sets is statistically significant or not, computed using Equation 18.


(18) t=Mean Difference/StandardError

The standard deviation (SD) quantifies the extent to which the performance measures deviate from the mean.

#### 4.5.3 Standard deviation (SD) formula

The standard deviation (SD) formula of a random variable, dataset, statistical population, or probability distribution is the square root of its variance, given in Equation 19.


(19) SD=sqr(1/N−1∗Σ(xi−x¯)^2)

#### 4.5.4 Effect size (Cohen’s d) formula

The effect size (Cohen’s d) formula is computed by taking the mean difference between two sets, and then dividing the result by the pooled standard deviation (SD), as presented in Equation 20.


(20) d=x¯1−x¯2/sqr(SD1^2+SD2^2)/2

**Figure 14:**
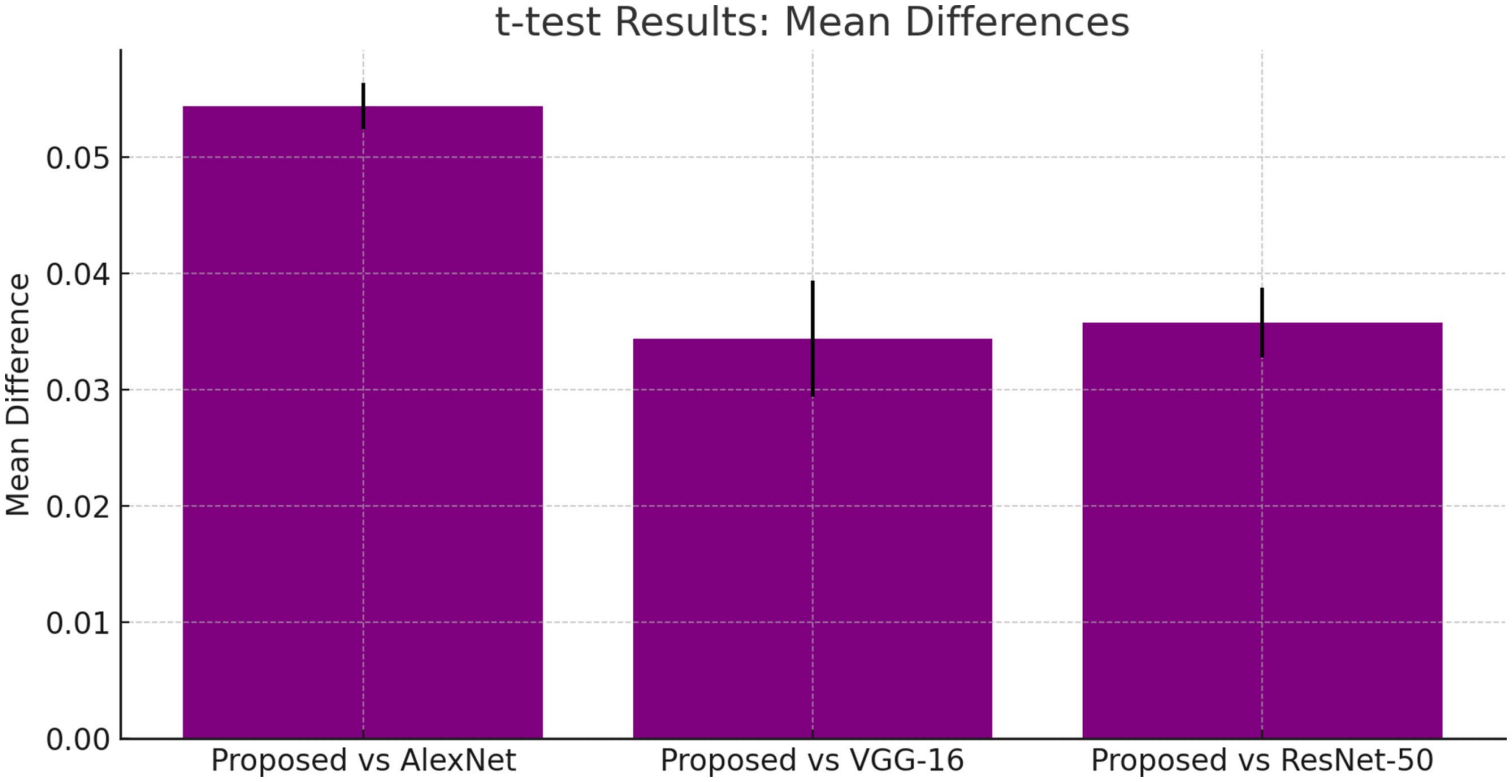
T-test results: mean differences.

**Figure 15:**
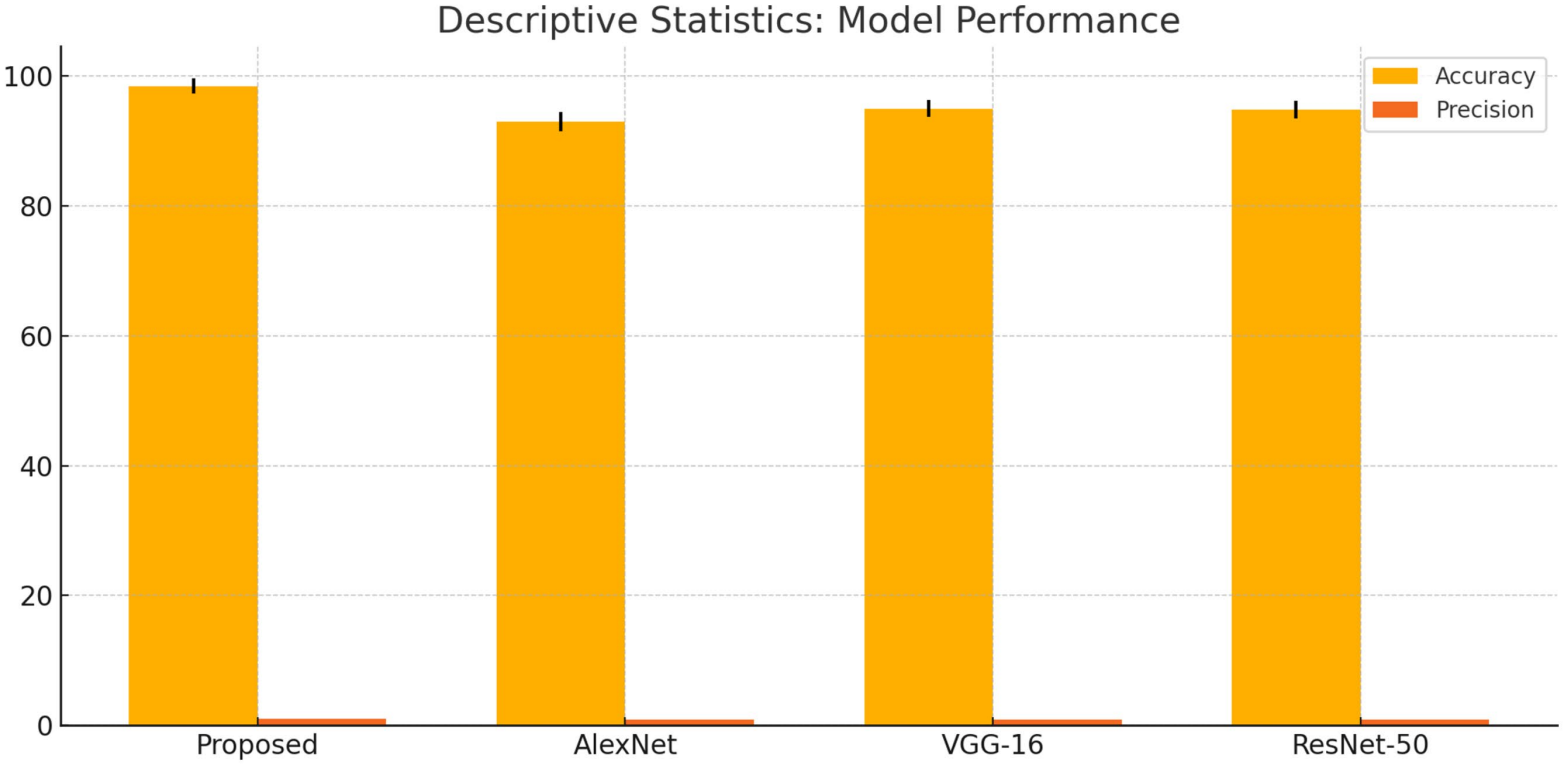
Descriptive statistics: model performance.

The suggested DCNN model outperforms AlexNet, VGG-16, and ResNet-50 in accuracy, precision, recall, and F1-score as shown in Figures 14, 15. The ANOVA and t-test results which also reflects in Table 8 of descriptive statistics give a thorough and realistic evaluation of the model’s clinical utility in diagnosing neurological illnesses.

**Table 8:** Descriptive statistics.

Model	Accuracy mean	Accuracy SD	Precision mean	Precision SD
Proposed	98.44%	1.2%	0.9849	0.01
AlexNet	93.00%	1.5%	0.930	0.02
VGG-16	95.00%	1.3%	0.945	0.015
ResNet-50	94.8%	1.4%	0.945	0.015

## 5 Conclusion and future work

This study proposed a novel Deep Convolutional Neural Network (DCNN) model for diagnosing neurological disorders, including Alzheimer’s, Parkinson’s, and seizure-related conditions, using MRI data. The model addressed challenges like class imbalance through data augmentation and weighted loss functions, which improved classification accuracy for underrepresented classes. It achieved high precision, recall, and F1-scores across all categories, including minority classes such as ‘moderate_demented’. The results demonstrated that the DCNN model outperformed existing methods, such as AlexNet and ResNet-50, in terms of diagnostic accuracy and efficiency. These findings highlight the potential of integrating advanced machine learning models into clinical workflows to enable faster and more reliable diagnoses, ultimately improving patient outcomes.

While the results are promising, this study also acknowledges certain limitations. The reliance on a single dataset may limit generalizability to real-world clinical scenarios with more diverse data distributions. Additionally, oversampling techniques, while effective, may introduce minor biases favoring minority classes. In the future work, we will focus on validating the model on external and multi-center datasets to ensure robustness across diverse populations; exploring advanced data augmentation techniques to simulate more realistic variability in medical imaging; incorporating multimodal data, such as clinical records with EEG to enhance diagnostic comprehensiveness and accuracy; as well as developing interpretable AI systems to provide clinicians with deeper insights into the model’s decision-making process. By addressing these areas, the proposed framework can be further refined and positioned as a robust tool for real-world neuro-diagnostic applications.

## Data Availability

Publicly available datasets were analyzed in this study. This data can be found at: https://www.kaggle.com/datasets/bilaliqbalai/new-alzheimer-mri12/data.

## Footnotes

^1^ https://www.kaggle.com/datasets/bilaliqbalai/new-alzheimer-mri12/data

## References

[1] Bayrak S Yucel E Takci H. Epilepsy radiology reports classification using deep learning networks. Computers, Materials & Continua. (2022) 70:3589–607. doi: 10.32604/cmc.2022.018742

[2] Erdaş ÇB Sümer E “A fully automated approach involving neuroimaging and deep learning for Parkinson’ s disease detection and severity prediction.” PeerJ Computer Science. (2023). 9:e1485. doi: 10.7717/peerj-cs.1485 PMC10403203 37547409

[3] Aminpour A Ebrahimi M Widjaja E. Lesion segmentation in Paediatric epilepsy utilizing deep learning approaches. Adv Artif Intell Mach Learn. (2022) 2:422–40. doi: 10.54364/AAIML.2021.1128

[4] Shivangi A.J Tripathi A., (2019). “Parkinson disease detection using deep neural networks,” *12th Int. Conf. Contemp. Comput. IC3 2019*, pp. 1–4.

[5] Hosseini-Asl E. Gimel’farb G. El-Baz A., “Alzheimer’s disease diagnostics by a deeply supervised adaptable 3D convolutional network,” no. 502, (2016). Available at: http://arxiv.org/abs/1607.00556 (Accessed July 15, 2024). 10.2741/4606 28930562

[6] Deivasigamani S Senthilpari C Yong WH. Machine learning method based detection and diagnosis for epilepsy in EEG signal. J Ambient Intell Humaniz Comput. (2021) 12:4215–21. doi: 10.1007/s12652-020-01816-3

[7] Zhang J Rao VM Tian Y Yang Y Acosta N Wan Z. Detecting schizophrenia with 3D structural brain MRI using deep learning. Sci Rep. (2023) 13:359. doi: 10.1038/s41598-023-41359-z 37660217 PMC10475022

[8] Gautam R Sharma M. Prevalence and diagnosis of neurological disorders using different deep learning techniques: a Meta-analysis. J Med Syst. (2020) 44:1519. doi: 10.1007/s10916-019-1519-7 31902041

[9] Taheri H Kaabouch N. A deep learning approach for diagnosis of mild cognitive impairment based on mri images. Brain Sci. (2019) 9:217. doi: 10.3390/brainsci9090217 31466398 PMC6770590

[10] Kalinaki K Malik O Ching D Lai C. International journal of applied earth observation and Geoinformation FCD-AttResU-net: an improved forest change detection in Sentinel-2 satellite images using attention residual U-net. Int J Appl Earth Obs Geoinf. (2023) 122:103453. doi: 10.1016/j.jag.2023.103453

[11] Rahman S Hasan M Sarkar AK Khan F. Classification of Parkinson’s disease using speech signal with machine learning and deep learning approaches. Eur J Electr Eng Comput Sci. (2023) 7:20–7. doi: 10.24018/ejece.2023.7.2.488

[12] Shoeibi A Khodatars M Ghassemi N Jafari M Moridian P Alizadehsani R. Epileptic seizures detection using deep learning techniques: a review. Int J Environ Res Public Health. (2021) 18:5780. doi: 10.3390/ijerph18115780 34072232 PMC8199071

[13] Anagun Y. Smart brain tumor diagnosis system utilizing deep convolutional neural networks. Multimed Tools Appl. (2023) 82:44527–53. doi: 10.1007/s11042-023-15422-w PMC10140727 37362644

[14] Talo M Yildirim O Baloglu UB Aydin G Acharya UR. Convolutional neural networks for multi-class brain disease detection using MRI images. Comput Med Imaging Graph. (2019) 78:101673. doi: 10.1016/j.compmedimag.2019.101673 31635910

[15] Irsheidat S. Duwairi R., (2020). “Brain tumor detection using artificial convolutional neural networks,” *11th Int. Conf. Inf. Commun. Syst. ICICS 2020*, pp. 197–203.

[16] Alkahtani H. Aldhyani TH Alzahrani MY, Deep learning algorithms to identify autismspectrum disorder in children-based facial landmarks. Applied Sciences. (2023). 13:4855.

[17] Mozhdehfarahbakhsh A Chitsazian S Chakrabarti P Chakrabarti T Kateb B Nami M. An MRI-based deep learning model to predict Parkinson’s disease stages. medRxiv. (2021) 2021:2081. doi: 10.1101/2021.02.19.21252081v1.abstract

[18] Chandaran SR Muthusamy G Sevalaiappan LR Senthilkumaran N. Deep learning-based transfer learning model in diagnosis of diseases with brain magnetic resonance imaging. Acta Polytech Hungarica. (2022) 19:127–47. doi: 10.12700/APH.19.5.2022.5.7

[19] Lee C van der Schaar MA. A deep learning approach for dynamic survival analysis with competing risks in CF. Pediatr Pulmonol. (2018) 53:261. doi: 10.1109/TBME.2019.2909027

[20] Pei X Chen L Guo Q Duan Z Pan Y Hou H. Materials & design robustness of machine learning to color, size change, normalization, and image enhancement on micrograph datasets with large sample differences. Mater Des. (2023) 232:112086. doi: 10.1016/j.matdes.2023.112086

[21] Kebaili A., “Deep learning approaches for data augmentation in medical imaging: a review,” Journal of Imaging. (2023). 9:81. 37103232 10.3390/jimaging9040081 PMC10144738

[22] Hitchens PL Morrice-West AV Whitton RC. Changes in thoroughbred speed and stride characteristics over successive race starts and their association with musculoskeletal injury data sources. Equine Vet J. (2023) 2022:194–204. doi: 10.1111/evj.13581 PMC10084173 35477925

[23] Kaur R Levy J Motl RW Sowers R Hernandez ME. Deep learning for multiple sclerosis differentiation using multi-stride dynamics in gait. IEEE Trans Biomed Eng. (2023) 70:2181–92. doi: 10.1109/TBME.2023.3238680 37819835

[24] Dogan Y. A new global pooling method for deep neural networks: global average of top-K max- pooling. Traitement du signal. (2023) 40:577–87. doi: 10.18280/ts.400216

[25] Reyad M Sarhan AM Arafa M. A modified Adam algorithm for deep neural network optimization. Neural Comput & Applic. (2023) 35:17095–112. doi: 10.1007/s00521-023-08568-z

[26] Langenberg B Janczyk M Koob V Kliegl R Mayer A. A tutorial on using the paired t test for power calculations in repeated measures ANOVA with interactions. Behav Res Methods. (2023) 55:2467–84. doi: 10.3758/s13428-022-01902-8 36002625 PMC10439102

[27] Nagaraja B Almeida F Yousef A Kumar P Ajaykumar AR Al-mdallal Q. Case studies in thermal engineering empirical study for Nusselt number optimization for the flow using ANOVA and Taguchi method. Case Stud Therm Eng. (2023) 50:103505. doi: 10.1016/j.csite.2023.103505

[28] Helaly HA Badawy M Haikal AY. Deep learning approach for early detection of Alzheimer’s disease. Cogn Comput. (2022) 14:1711–27. doi: 10.1007/s12559-021-09946-2 PMC8563360 34745371

[29] Yin W Mostafa S Wu FX. Diagnosis of autism Spectrum disorder based on functional brain networks with deep learning. J Comput Biol. (2021) 28:146–65. doi: 10.1089/cmb.2020.0252 33074746

[30] di F la F Petrone V Battaglia S Orlandi S Ippolito G. Accuracy of EEG biomarkers in the detection of clinical outcome in disorders of consciousness after severe acquired brain injury: preliminary results of a pilot study using a machine learning approach. Biomedicine. (2022) 10:897. doi: 10.3390/biomedicines10081897, PMID: 36009445 PMC9405912

[31] Alotaibi G Awawdeh M Farook FF Aljohani M. Artificial intelligence (AI) diagnostic tools: utilizing a convolutional neural network (CNN) to assess periodontal bone level radiographically — a retrospective study. BMC Oral Heal. (2022) 22:1–7. doi: 10.1186/s12903-022-02436-3 PMC9469589 36100856

